# Rare Case of Small Bowel Obstruction Secondary to Cryptosporidium in a Young Patient With Uncontrolled AIDS

**DOI:** 10.7759/cureus.16040

**Published:** 2021-06-29

**Authors:** Anton Mararenko, Steven Douedi, Abbas Alshami, Mihir Odak, Swapnil V Patel

**Affiliations:** 1 Internal Medicine, Jersey Shore University Medical Center, Neptune, USA

**Keywords:** small bowel obstruction, cryptosporidium, human immunodeficiency virus, acquired immune deficiency syndrome, bowel wall edema

## Abstract

Small bowel obstruction is a common cause of abdominal pain and accounts for approximately 20% of surgical admissions related to abdominal pain. In the United States alone, there are over 300,000 admissions annually for small bowel obstruction and account for every 15 out of 100 admissions for abdominal pain. If treated appropriately with medical management, over 80% of cases resolve without life-threatening, long-term complications or the need for surgical intervention. The three most common causes including adhesions, tumors, and hernias account for the majority of cases. Less frequently reported causes include infections. We present the case of a 26-year-old male with a history of AIDS who was found to have a small bowel obstruction in the setting of active *Cryptosporidium* infection. *Cryptosporidium* is an opportunistic infection that more commonly affects immunocompromised hosts, especially those noncompliant with antiretroviral therapy. Our patient had an uncomplicated hospital course and made a full recovery due to early diagnosis and immediate intervention. We hope to make the medical community more aware of this rare and potentially life-threatening association given the rarity of such a presentation. Early diagnosis and intervention are critical to preventing morbidity and mortality.

## Introduction

Small bowel obstruction is a commonly encountered clinical condition requiring both medical and surgical attention. Small bowel obstruction can be described anatomically by a transition point, or point of obstruction, that results in proximal bowel dilation as well as distal bowel decompression [1]. The most common causes of small bowel obstruction in developed countries include intra-abdominal adhesions, which make up approximately 65-75% of cases, followed by hernias, malignancy, inflammatory bowel disease, and volvulus occurring collectively in 18-38% of cases [1]. In the developing world, the most common causes include hernias (30%), adhesions (30%), and tuberculosis (10%) [2]. Early diagnosis of small bowel obstruction is critical as up to 25% of patients admitted with small bowel obstruction require surgical intervention if untreated [2]. We present the case of a 26-year-old male with AIDS who was found to have a small bowel obstruction in the setting of active *Cryptosporidium* infection. *Cryptosporidium*, an opportunistic pathogen, can infect both immunocompetent and immunosuppressed hosts. Although it is well known to be the most common parasitic cause of infectious diarrhea in immunocompromised hosts, its association with small bowel obstruction has been rarely reported in adult patients with HIV or AIDS. There have been case reports linking small bowel obstruction with disseminated histoplasmosis; however, it has not been associated with *Cryptosporidium* infection [3].

## Case presentation

A 26-year-old male with a past medical history significant for HIV complicated by AIDS presented to the emergency department after being found to have abnormal routine blood work as an outpatient. The patient acquired HIV through vertical transmission at birth and was largely noncompliant with medical therapy throughout his life. His antiretroviral regimen consisted of bictegravir-emtricitabine-tenofavir (50-200-25 mg) and sulfamethoxazole-trimethoprim for primary prophylaxis. The most recent CD4+ T-cell count was 3 cells/uL (reference: 430-1800 cells/uL) and an HIV viral count of 4,570,000 copies. The extent of noncompliance was also evident by previous admissions for *Pneumocystis* pneumonia infection and frequent clinic visits for oral pain secondary to oral thrush.

Upon admission, the patient reported that his only complaints included decreased oral intake, nausea, vomiting, and intermittent episodes of loose bowel movements of two-day duration. His vitals on admission were a heart rate of 87 beats per minute, respiratory rate of 18 breaths per minute, oxygen saturation of 100% on room air, blood pressure of 90/52 mmHg, and temperature of 97.6°F measured orally. Physical examination was significant for a cachectic appearance and coarse breath sounds in both lung fields. Otherwise, the abdominal examination was benign as there was no tenderness, rigidity, or guarding. Laboratory findings, as illustrated in Table 1, were significant for several electrolyte derangements and a high anion gap metabolic acidosis (HAGMA). As lactic acid was within normal levels on admission, the HAGMA was likely due to starvation ketoacidosis. The patient was also found to have an acute kidney injury with a creatinine of 1.36 mg/dL on presentation versus a baseline of 0.48-0.68 mg/dL over the last year. The patient was admitted to the medical service for further workup and management. He was treated with aggressive electrolyte repletion. The hyponatremia was improving appropriately with intravenous normal saline infusion and the remaining electrolytes remained stable. He was also resumed on antiretroviral treatment with bictegravir-emtricitabine-tenofavir (50-200-25 mg) and a prophylactic dose of sulfamethoxazole-trimethoprim given the severe underlying immunosuppression in the setting of noncompliance.

**Table 1:** Focused laboratory data from metabolic panel and blood counts. ALT: alanine aminotransferase; AST: aspartate transaminase; BUN: blood urea nitrogen; eGFR: estimated glomerular filtration rate, race adjusted

	Reference range	Patient’s value
BUN	5-25 mg/dL	29 mg/dL
Creatinine	0.61-1.24 mg/dL	1.36 mg/dL
eGFR	>60 mL/min/1.73 m^2^	>60 mL/min/1.73 m^2^
Sodium	136-145 mmol/L	119 mmol/L
Potassium	3.5-5.2 mmol/L	2.5 mmol/L
Serum chloride	96-110 mmol/L	85 mmol/L
Anion gap	5-13 mmol/L	14 mmol/L
Carbon dioxide	24-31 mmol/L	20 mmol/L
Magnesium	1.3-2.5 mg/dL	1.5 mg/dL
Alkaline phosphatase	38-126 U/L	98 U/L
Total bilirubin	0.2-1.3 mg/dL	0.9 mg/dL
AST	10-42 U/L	25 U/L
ALT	10-60 U/L	34 U/L
White blood cells	4,500-11,000/uL	6,800/uL
Hemoglobin	13.2-17.5 g/dL	13.0 g/dL
Hematocrit	40.0-53.0%	37.4%
Mean corpuscular volume	80.0-100.0 fL	79.2 fL
Platelet count	140,000-450,000/uL	418,000/uL

**Figure 1:**
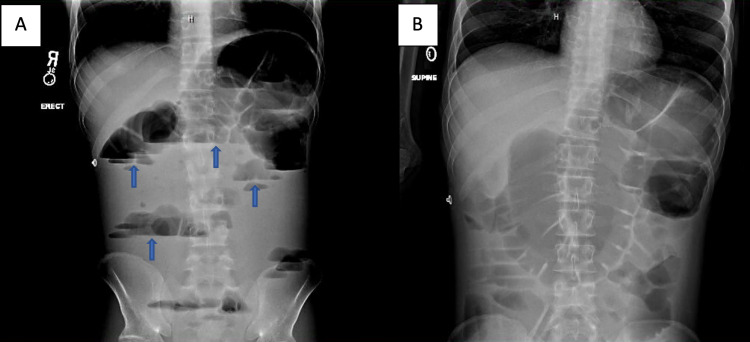
Abdominal anterior-posterior X-ray film. (A) and (B) demonstrate signs of small bowel obstruction with extensive dilations of the bowel and numerous air-fluid levels (marked by blue arrows). No signs of air are appreciated in the large intestine.

The patient’s hospital course acutely worsened on day two as nausea and vomiting had become refractory to antiemetic therapy including oral trimethobenzamide and ondansetron. Intravenous antiemetic therapy was limited as the patient had significant prolongation of the QTc interval on electrocardiograms. Nausea and vomiting had evolved to several loose bowel movements. An immediate abdominal X-ray was done that confirmed small bowel obstruction (Figure 1). Intravenous hydration therapy was continued, and a nasogastric tube was placed for bowel decompression. After 24 hours of nasogastric suctioning, a small bowel series with Gastrografin was performed which showed that the obstruction had resolved. A gastrointestinal pathogen panel ruled out the presence of *Shigella*, *Campylobacter*, *Salmonella*, *Giardia *species, and Shiga toxins 1 and 2 by polymerase chain reaction. *Clostridium difficile* testing was also negative. Stool cultures were significant only for normal microflora. Interestingly, *Cryptosporidium* was detected by antigen testing. The laboratory data corresponded with infection as the white blood cell count remained stable; however, the bandemia worsened from normal on admission to 36% (reference range: 5-11%) at the peak of his symptoms.

The patient responded appropriately to the standard of care therapy for small bowel obstruction. His symptoms had improved within 24-48 hours after the initiation of nasogastric suctioning, and he reported significant improvement and resolution of symptoms. He was able to tolerate a diet and was safely discharged to a rehabilitation facility.

## Discussion

Small bowel obstruction is a commonly encountered medical problem in the United States. Approximately 15 out of every 100 admissions citing abdominal pain are secondary to small bowel obstruction and collectively account for over 300,000 admissions annually [4]. The diagnosis of small bowel obstruction is often a clinical diagnosis that is confirmed by imaging studies. Early signs of intestinal obstruction manifest as nausea, intolerance of oral intake, vomiting, loose bowel movements, and abdominal tenderness with guarding without another clear diagnosis. Auscultation of localized and high-pitched bowel sounds is another clinical hint suggestive of obstruction. Abdominal X-ray is often the first imaging test performed; however, the sensitivity ranges 60-93% and depends heavily on the interpretation by the reading radiologist [5]. When small bowel obstruction is suspected but X-ray imaging is inconclusive, a CT is indicated to help delineate the diagnosis. On CT imaging, small bowel obstruction is defined by a dilation of the small bowel to >2.5 cm with distal decompression beyond the transition point [6].

In some cases, the primary team may elect to perform a small bowel series with diatrizoate meglumine, better known as Gastrografin, as it is a radio-opaque contrast medium that can help identify points of intraluminal obstruction. Diatrizoate meglumine has been associated with a significantly decreased in-hospital length of stay by nearly two days [7]. The efficacy can be partially explained by the molecular properties of the compound. It is an ionic solution with a high osmolarity that, theoretically, can increase the oncotic pressure within the intestinal luminal and reduce small bowel wall edema [8]. Despite the efficacy in symptomatic relief, it has not been associated with a decreased rate of overall complications, mortality rates, or surgical outcomes. Furthermore, its use may be limited as it has been reported to be fatal if aspirated [9].

Although the etiology of small bowel obstruction is broad, over 80% of cases are due to intra-abdominal adhesions, tumors, and hernias. Less common causes include intra-abdominal inflammation and infection, intestinal stricture, volvulus, gallstones, intussusception, superior mesenteric artery syndrome, and congenital anomalies of the gastrointestinal tract. Regardless of the etiology, the management remains largely the same and consists of intravenous fluid hydration, nasogastric suctioning to promote decompression, management of electrolyte derangements, and prohibiting any oral intake until obstruction resolves. Early diagnosis and intervention are critical as the majority of cases resolve without surgical intervention and have a much lower risk of associated complications [2]. The most severe complications of small bowel obstruction include tissue ischemia, necrosis, infection due to intestinal stasis, and perforation [10]. If the obstruction occurs in the proximal small bowel, the prolonged stasis can result in an overgrowth of microflora in an otherwise sterile portion of the gut and result in aspiration of feculent material.

Our patient was a young male without any comorbidities or surgical history aside from untreated AIDS. The cause of small bowel obstruction in our patient is unclear; however, it is likely related to the acute gastrointestinal infection caused by *Cryptosporidium*. *Cryptosporidium *is a parasitic protist normally known to infect birds, reptiles, amphibians, and fish [11]. The two most commonly described species that infect humans include *C. parvum* and *C. hominis*. Transmission of the parasite often includes direct oral consumption of contaminated water [12]. Once in the small intestine, the spindle-shaped parasite can freely travel and infect enterocytes. The likely pathophysiology of obstruction in our patient involved bowel wall edema with stasis of reactive phlegmon. Localized inflammation of lymph nodules may have induced intussusception or volvulus; however, given the imaging findings and presentation, this is highly unlikely.

At the time of writing, there are no reported cases of small bowel obstruction in adult patients with AIDS and active *Cryptosporidium *infection. An extensive report was provided by Suh et al. in which they described 23 patients with AIDS who were found to have gastrointestinal histoplasmosis. However, only three of those patients were found to have a small bowel obstruction [13]. Despite the prevalence of *Cryptosporidium *among patients with HIV, it has not been reported in adult patients to date. We hope to make the medical community more aware of the possible association between active *Cryptosporidium *infection and small bowel obstruction. Clinical awareness of the potential correlation can lead to early diagnosis and intervention, thus preventing life-threatening complications of small bowel obstruction.

## Conclusions

Small bowel obstruction remains a common cause of admission requiring both medical and surgical evaluation. Abdominal X-ray and CT imaging remain the ideal imaging modalities of choice to confirm the clinical diagnosis. A small bowel series with Gastrografin has the added benefit of introducing a high osmolar medium that can potentially decrease bowel wall edema while providing useful diagnostic information. Most cases of small bowel obstruction can be traced to abdominal adhesions, tumors, or adhesions. Infectious causes can be seen more commonly in immunosuppressed populations such as those with HIV. *Cryptosporidium *is an opportunistic pathogen and is the most common parasitic cause of infectious diarrhea in patients with AIDS. Our patient had an uncomplicated hospital course due to early diagnosis and intervention. The pathophysiology of obstruction in our patient who had no history of abdominal surgery or risk factors is likely due to bowel wall edema as well as intestinal phlegmon that resulted in mechanical obstruction. We hope to make the medical community more aware of the possible association of *Cryptosporidium *infection resulting in small bowel obstruction as the course can be rapidly progressive and life-threatening. If treated promptly and early in the disease course, most cases of small bowel obstruction resolve without requiring surgical intervention.
